# Changes in Protein Composition in the Grain and Malt after Fusarium Infection Dependently of Wheat Resistance

**DOI:** 10.3390/pathogens8030112

**Published:** 2019-07-26

**Authors:** Valentina Spanic, Daniela Horvat, Georg Drezner, Zvonimir Zdunic

**Affiliations:** 1 Department of Small Cereal Crops, Agricultural Institute Osijek, Osijek, Juzno predgradje 17, 31000 Osijek, Croatia; 2 Agrochemical Laboratory, Agricultural Institute Osijek, Osijek, Juzno predgradje 17, 31000 Osijek, Croatia; 3 Department of Maize, Agricultural Institute Osijek, Agricultural Institute Osijek, Osijek, Juzno Predgradje 17, 31000 Osijek, Croatia

**Keywords:** albumins, globulins, gliadins, glutenins, malt, wheat

## Abstract

The grain yield, as well as the quality and safety of the wheat grains and corresponding malt can be compromised by *Fusarium* spp. infection. The protein content of the grain affects the chemical composition and enzyme levels of the finished malt. The malting industry demands varieties with good malting and brewing performance, as well as good agronomic performance and disease resistance. The best method of disease control is breeding and selection for resistant varieties. Due to higher requirements for malting wheat worldwide, the goal of this investigation was to explore changes in protein distribution in wheat grains and corresponding malt, which are under higher pressure of *Fusarium* head blight (FHB) infestation in field conditions. The present study provides new knowledge on the impact of the FHB on the distribution of protein components of naturally *Fusarium*-infected (control) and *Fusarium-*inoculated wheat varieties in the grain and the corresponding malt in two consecutive years (2015/2016 and 2016/2017). The results showed that *Fusarium* infection of the susceptible variety Golubica, decreased total glutenins (5.9%), and both high and low molecular weight glutenin subunits (2.5% and 3.5%, respectively) in wheat grains, compared to control, in 2016. In contrast, gliadins and α-gliadins increased significantly (+7.6% and +5.1%, respectively) in the same variety. Wheat grains of the more resistant variety Vulkan showed an increase of the total glutenins content (+4.3%), and of high and of low molecular weight glutenin subunits (+1.2% and +3.2%, respectively) after *Fusarium*-inoculation, compared to naturally infected grains in 2016. Susceptible variety Golubica increased total glutenins (+9.1%), and both high and low molecular weight glutenin subunits (+3.5% and +5.6%, respectively) after *Fusarium*-inoculation in wheat malt, compared to naturally infected malt in 2016. In 2017, when disease pressure was higher than in 2016, there was a tendency in all varieties to increase gliadins and its sub fractions after malting, and to decrease glutenins and its sub fractions in *Fusarium*-inoculated treatment. In conclusion, FHB dramatically depressed grain yield (up to 37%) and quality (glutenins and high molecular weight subunits) in the susceptible *Fusarium* variety, which makes it inconvenient for malting.

## 1. Introduction

*Fusarium* head blight (FHB) affects heads of the wheat and severe infection decreases grain yield and quality. Moreover, FHB can have a negative impact on the malting process. It was previously concluded that wheat proteins influence brewing because during malting a significant amount of the cereal proteins are hydrolyzed and therefore become water-soluble [1,2]. In general, wheat grain proteins are divided into metabolic non-gluten (albumins and globulins) and storage proteins (gliadins and glutenins). Gliadins have been classified as α-, β-, γ- and ω-gliadins with similar amino acid sequences. High molecular weight and low molecular weight glutenin subunits contain alcohol-insoluble polymeric proteins which can be often degraded by *Fusarium* infection. Moreover, FHB affects the grain protein content [3]. This aspect has a negative impact on the malting process, i.e., excessive grain proteins are undesirable because they are associated with lower malt quality and extract levels [4]. Utilization of resistant wheat varieties is the best method to control FHB and to prevent technological damages of the grain [5,6,7].

Barley heads infected with *Fusarium* spp. can influence the final malt and beer as well as beer consumers’ health, due to mycotoxin contamination [8]. In addition, the presence of *Fusarium* spp. in barley kernels is related to gushing [9]. Therefore, barley with high concentrations of deoxynivalenol (DON) is rejected for malting and beer production [10] and is instead used as feed or in biogas/bioethanol production. DON is one of several mycotoxins produced by certain *Fusarium* species that frequently infect wheat in the field or during storage [11].

The malting process consists of steeping the grain in the water which will lead to germination in controlled conditions. Afterwards, green malt is subjected to kilning (drying) at gradually increased temperatures. During steeping and germination, hydrolytic enzymes will breakdown the endosperm cell walls, which results in a source of sugars, degradable starch, amino acids, and enzymes. Therefore, studies of protein content during technological stages are of interest [12].

The aim of our study was to extend the research of protein distribution in wheat malt from naturally *Fusarium*-infected and *Fusarium*-inoculated grains in a two-year field experiment where resistant and susceptible varieties were compared. In addition, agronomical, qualitative, and physical characteristics were measured. The utilization of resistant wheat varieties in disease management programs and the malting industry can provide an effective, safe, and sustainable means to control *Fusarium* disease. To our knowledge, protein distribution has not been previously investigated in wheat malt, as the result of the cumulative impact of the FHB species complex in naturally and artificially infected wheat.

## 2. Results and Discussion

Even if *Fusarium* spp. severity in inoculated treatment changed throughout the considered years, all the analyzed wheat varieties were revealed to be artificially infected by these fungal species, with some varieties showing higher susceptibility with respect to others based on disease severity. The ‘variety (V)’, ‘treatment (T)’, ‘year (Y)’ and ‘malt/grain (MG)’ effect was always significant and explained the highest proportion of data variability except for of γ-gliadins in different years (Supplementary Material 1). All interactions were significant except for % of albumins and % γ-gliadins in the ‘malt/grain by treatment’, % of ω- and γ-gliadins in the ‘malt/grain by treatment by variety’ interaction (data not shown). Since the weather conditions varied greatly between these two years, this explains the strong influence of the year. While for albumins and globulins the effect of the treatment was relatively small, the influence of variety was the largest among other traits of protein distribution. ANOVA indicated that the year grown affected glutenins more than other protein components, which gave us indication that glutenins seemed to be more variable due to environmental changes then other components. It is well documented that the gluten protein composition can be influenced either by genetic [13] or environmental factors [14,15]. *Fusarium* head blight (FHB) severity varied across the years (Table 1). In general, FHB severity increased linearly during the time-course experiment. Overall, the area under disease progress curve (AUDPC) for disease severity per variety in 2016 ranged from 18.3 (Vulkan) to 114.5 (Golubica), while AUDPC disease severity per variety in 2017 ranged from 72.5 (Olimpija) to 430.0 (Golubica). The first rating was performed once the first symptoms appeared at 18 days post inoculation (dpa) in 2016, and at 10 dpa in 2017. Furthermore, the FHB severity was higher in 2017 compared with 2016 for all wheat varieties. In 2016, Olimpija and Kraljica were moderately resistant, with the *Fusarium* severity per plot at the last sampling point at 18% and 20%, respectively. At the last sampling point in 2017, Vulkan, Olimpija and Kraljica were moderately resistant, with scores of 20%, 18% and 20%, respectively. (Table 1). 

### 2.1. Protein Distribution in the Grain and Malt Dependently of Control and Inoculation

**Figure 1:**
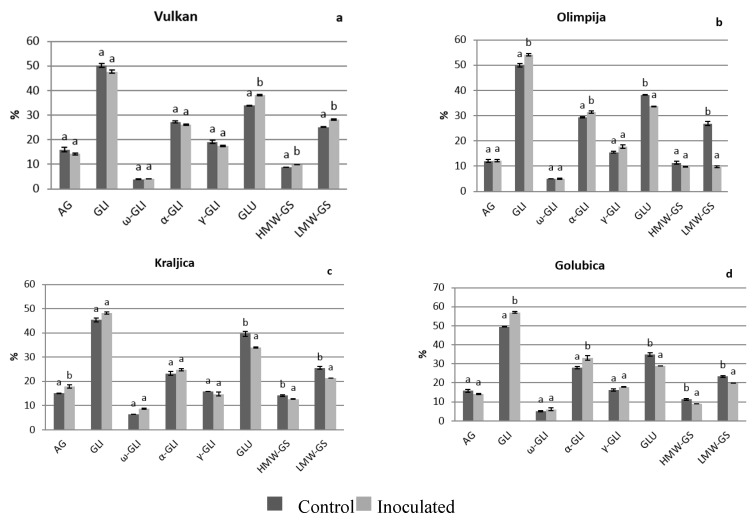
Protein components in the wheat grain of Vulkan (**a**); Olimpija (**b**); Kraljica (**c**) and Golubica (**d**) in two treatments. Values are means±SE from two independent trials in 2016. Different letters indicate significant differences (LSD test with *p* = 0.05) in naturally infected and artificially inoculated plants. a, b—Different letters mean different statistical differences in naturally infected or artificially inoculated treatment for each variety; AG—Albumins and globulins, GLI—Gliadins, GLU—Glutenins; HMW-GS—High molecular weight-glutenin subunits, LMW-GS—Low molecular weight-glutenin subunits.

**Figure 2:**
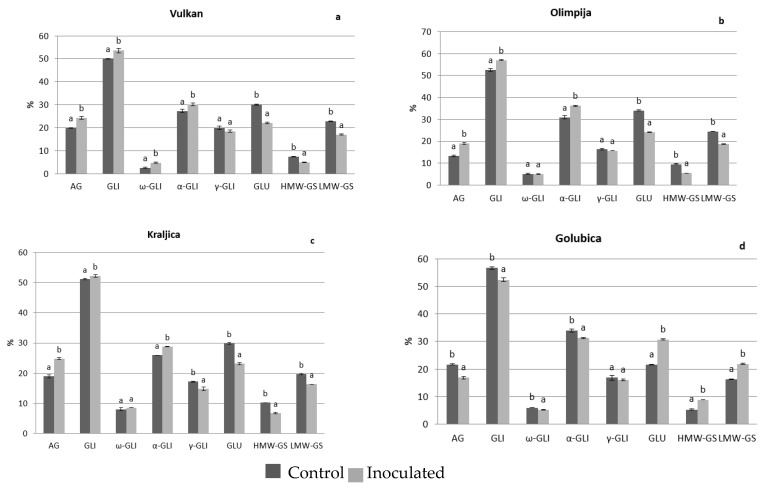
Protein components in the wheat malt of Vulkan (**a**), Olimpija (**b**), Kraljica (**c**) and Golubica (**d**) in two treatments. Values are means ±SE from two independent trials in 2016. Different letters indicate significant differences (LSD test with *p* = 0.05) in naturally infected and artificially inoculated plants. a, b—Different letters mean different statistical differences in naturally infected or artificially inoculated treatment for each variety; AG—Albumins and globulins, GLI—Gliadins, GLU—Glutenins; HMW-GS—High molecular weight-glutenin subunits, LMW-GS—Low molecular weight-glutenin subunits.

**Figure 3:**
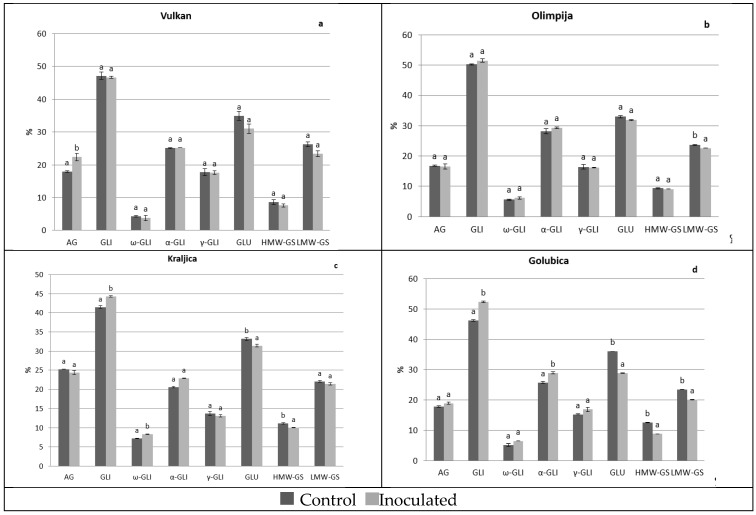
Protein components in the wheat grain of Vulkan (**a**), Olimpija (**b**), Kraljica (**c**) and Golubica (**d**) in two treatments. Values are means ± SE from two independent trials in 2017. Different letters indicate significant differences (LSD test with *p* = 0.05) in naturally infected and artificially inoculated plants. a, b—Different letters mean different statistical differences in naturally infected or artificially inoculated treatment for each variety; AG—Albumins and globulins, GLI—Gliadins, GLU—Glutenins; HMW-GS—High molecular weight-glutenin subunits, LMW-GS—Low molecular weight-glutenin subunits.

**Figure 4:**
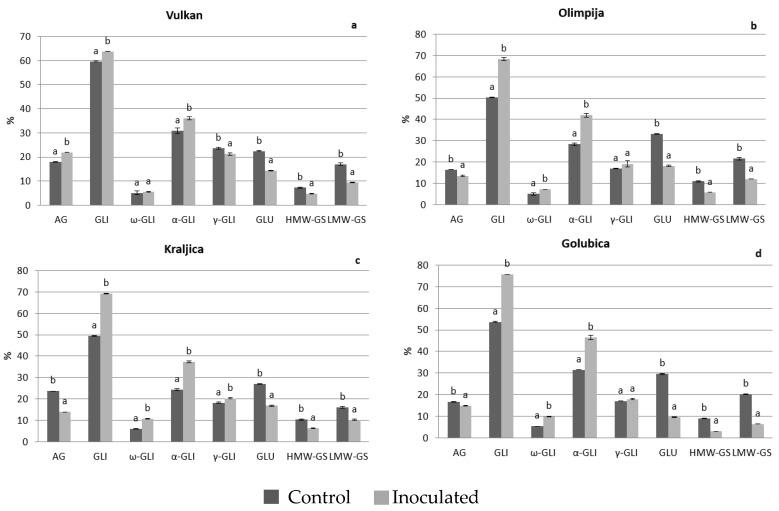
Protein components in the wheat malt of Vulkan (**a**), Olimpija (**b**), Kraljica (**c**) and Golubica (**d**) in two treatments. Values are means±SE from two independent trials in 2017. Different letters indicate significant differences (LSD test with *p* = 0.05) in naturally infected and artificially inoculated plants. a, b—different letters mean different statistical differences in naturally infected or artificially inoculated treatment for each variety; AG—Albumins and globulins, GLI—Gliadins, GLU—Glutenins; HMW-GS—High molecular weight-glutenin subunits, LMW-GS—Low molecular weight-glutenin subunits.

In 2016, there were not any significant differences between albumins and globulins in naturally infected and *Fusarium*-inoculated treatment for grains of Vulkan (the most FHB resistant variety in 2016) and Olimpija (moderately FHB resistant variety in 2016) (Figure 1a,b). Kraljica (moderately FHB resistant variety in 2016) increased albumins and globulins in inoculated treatment, compared to naturally infected (Figure 1c) and Golubica (FHB susceptible variety in 2016) remained albumins and globulins at the same significant level (Figure 1d). Olimpija had the lowest values of albumins and globulins in the naturally infected grains by far compared to other varieties (data not shown). An increasing trend occurred in malt in 2016, where in addition to Kraljica, both Olimpija and Vulkan increased albumins and globulins in *Fusarium*-inoculated treatment (Figure 2a–d). The exception was Golubica, which showed decreased albumins and globulins after *Fusarium* inoculation, compared to natural infection (Figure 2d). In 2017, in both grains and malt, Vulkan (moderately FHB resistant in 2017) increased albumins and globulins after *Fusarium* inoculation (Figure 3a and Figure 4a). The other three varieties did not significantly change albumins and globulins in the grains in inoculated treatment, compared to naturally infected treatment (Figure 3b–d). After malting, albumins and globulins were decreased in those varieties in *Fusarium*-inoculated treatment (Figure 4b–d). The highest values of albumins and globulins in malt inoculated samples in 2016 were for Vulkan and Kraljica. Albumins and globulins as a soluble protein could be important for the yeast growth in fermentation and also in malt beer color development. The increase ratio of the albumins and other soluble proteins and the decrease ratio of the glutenins were found to have a significant positive correlation with the Kolbach index, and the corresponding r-values were 0.883 and 0.975 [10].

In 2016, Olimpija and Golubica experienced significantly increased gliadins and α-gliadins in *Fusarium*-inoculated grains, compared to naturally infected grains (Figure 1b,d), as well as gliadins and ω- and α-gliadins in the malt (Vulkan) and gliadins and α-gliadins (Olimpija) after malting (Figure 2b). Kraljica and Golubica decreased glutenins, high and low molecular weight subunits. Also, Olimpija decreased glutenins and low molecular weight subunits, in inoculated grains, compared to naturally infected grains, with exception of FHB resistant variety Vulkan which increased glutenins, high and low molecular weight subunits in *Fusarium* infection. Similar increase in resistant variety was obtained by Spanic et al. [16]. Only Golubica (FHB susceptible variety) increased glutenins, high and low molecular weight subunits in inoculated malt samples, compared to naturally infected malt (Figure 2d). Other three varieties decreased those parameters in *Fusarium* infection (Figure 2a–c). Interestingly, Vulkan had the lowest glutenins, high molecular weight subunits in naturally infected grain samples, as well as Golubica in naturally infected malt among other varieties, which both increased glutenins and high molecular weight subunits.

In 2017, in *Fusarium*-inoculated grains, Kraljica increased gliadins and ω-gliadins, and Golubica gliadins and α-gliadins. But those varieties had significantly decreased glutenins and its sub fractions in inoculated grains, compared to naturally infected grains (except low molecular weight subunits for Kraljica) (Figure 3c,d). After malting, all varieties had tendency to increase gliadins and its sub-fractions (except α- and γ- for Vulkan). Also, a significant decrease of glutenins, high and low molecular weight subunits was observed among all four varieties in *Fusarium*-inoculated samples, compared to naturally infected (Figure 4a–d). Similarly in previous investigations, the wheat glutenin fractions and types were found to be more strongly affected by the *Fusarium* spp. [17]. The significantly lowest glutenins, high and low molecular weight subunits had Golubica in inoculated treatment of malt, similarly as in inoculated treatment of grains in 2017. The increase of gliadin content is explained by the degradation of glutenin sub fractions by *Fusarium* proteases [18], which was the case in current research and particularly pronounced in Golubica in both years where glutenins, high and low molecular weight subunits were significantly reduced in *Fusarium*-inoculated treatment, compared to naturally infected treatment both in the grains and malt. A similar increase of total gliadins content and all gliadins sub fractions and decrease of glutenins in inoculated wheat, compared to naturally infected was observed by Eggert et al. [17] and Horvat et al. [3]. It is very interesting that the FHB resistant variety Vulkan showed opposite tendency of increase of glutenins, high and low molecular weight subunits in *Fusarium*-inoculated grains. *Fusarium* spp. has been shown to be aggressive invaders of wheat kernels and are known to invade the endosperm, leading to degradation of gluten proteins by secreting proteolytic enzymes. Also, in this research, higher *Fusarium* spp. infection reduced glutenins. Similarly, in the research of Horvat et al. [3] the highest *Fusarium* affected protein components were glutenins, high and low molecular weight subunits. According to Eggert et al. [18] gluten digestion by F. graminearum proteases occurred while the high molecular weight sub fraction was the mostly affected. High molecular weight sub fractions are known to be important for dough properties and baking volume of the bread. It is well known that glutenins are synthesized more rapidly than gliadins during the later stages of the kernel maturation process [19] and possibly accumulation of glutenins could be incomplete if forced maturity occurs due to weather conditions with gliadins escaping disease.

During process of malting larger molecules (proteins and carbohydrates) are broken and utilized by germination shoots and roots and due to that reduction in the protein content occurred in barley during malting [20]. Guo et al. [21] indicated that the wheat protein content has to be in the range of 12.72%–13.88% for obtaining satisfactory wheat malt quality and the insoluble/soluble protein ratio should be in the range of 1.44–2.23. In 2016, after malting, the situation completely changed for the FHB resistant variety-Vulkan and two moderately FHB resistant varieties-Olimpija and Kraljica, with all three indicating significantly increased albumins and globulins and reduced high and low molecular weight subunits in *Fusarium*-inoculated malt, compared to naturally infected malt. In 2017, Vulkan increased albumins and globulins in both *Fusarium*-inoculated grains and malt. In the research of Osman et al. [1] malting was the most effective process remarkably increasing the soluble protein contents in barley, but in non-inoculated grains. According to Faltermaier et al. [2], during malting, proteins were hydrolyzed and therefore became water-soluble. In our research, on the other side, in *Fusarium*-inoculated malt, compared to naturally infected malt, all wheat varieties had tendency to increase gliadins and some sub fractions and to reduce high and low molecular weight subunits (Figure 4a–d). This is in accordance to research of Oliveira et al. [22] who showed that *Fusarium* spp. interfered with the grain hydrolytic protein profile, thereby altering the grain’s protein content and quality, during malting. According to Sarlin et al. [23] it is likely that more of the fungal proteinases were synthesized during the grain germination in barley which hydrolyzed cereal storage proteins. 

Since *Fusarium* infection of wheat in this study was provoked by artificial inoculation, it was assumed that *Fusarium* severity in this treatment will be higher than in naturally infected plants. In 2016 and 2017, Golubica (FHB-susceptible variety) significantly reduced glutenins, high and low molecular weight subunits in *Fusarium*-inoculated grains, compared to naturally infected grains, while in 2016, Vulkan increased glutenins, high and low molecular weight subunits. In 2017, Vulkan did not show any significant changes in those parameters. These protein fractions are the main responsible protein components for the quality of wheat dough properties. 

As consequence, after malting there was a reduction in glutenins and high molecular weight subunits in *Fusarium*-inoculated grain samples, compared to some varieties that were naturally infected, such as in FHB-resistant variety Vulkan in both 2016 and 2017. In more infected grains this is not the case [24], as we could observe in the Golubica-FHB susceptible variety, which in malt in 2016 increased glutenins or high molecular weight subunits in infected samples, compared to naturally infected samples. 

### 2.2. Grain Yield and Other Agronomical Traits

Determination of the grain yield and other agronomical and physiological characters has been analyzed in two consecutive years of the research, and for this reason those traits were introduced later in the manuscript, as a tool for fulfilling broader image about FHB losses in some important traits during *Fusarium* attack and its relation with protein composition after malting. Relative losses in wheat grain yield due to the effect of FHB ranged from 0% (Kraljica in 2016) to 56.2% (Golubica in 2017) (Table 2). The physical characteristics of kernel, meaning the test weight and 1000 kernel weight in *Fusarium*-inoculated treatment, resulted in a decrease of these two traits, compared to during naturally infected treatment (except for Kraljica in 2016). Mostly pronounced decrease was in Golubica in both years for the test weight (15.4%). Highly susceptible FHB variety had grain yield losses in *Fusarium*-inoculated treatment, compared to being naturally infected, as consequence of losses in test weight and 1000 kernel weight in 2017, as components of the yield. Common decrease of grain yield and test weight in correlation with increase of *Fusarium* severity was previously reported by many researchers [25,26].

According to different losses for grain yield in *Fusarium*-inoculated treatment, compared to naturally infected, susceptible wheat variety does not possess type V *Fusarium* resistance (yield tolerance), trait which was suggested by Mesterhazy et al. [27]. Also, the spike length was reduced by *Fusarium* infection up to 17.4% with reduced glutenins, high and molecular weight subunits for Golubica in 2016. Vulkan showed the opposite tendency, involving increased spike length with increased glutenins, high and low molecular weight subunits in 2016. Protein content in all wheat varieties in 2016 was higher in *Fusarium*-inoculated treatment then in naturally infected, while in 2017 Vulkan and Olimpija increased protein content in naturally infected treatment, compared to *Fusarium*-inoculated treatment. Also, starch value was affected up to 7.1% in Olimpija in 2017 (Table 2). Golubica increased starch in *Fusarium*-inoculated grains along with gliadins and ω-gliadins. Pathogens of the FHB complex impacted negatively on the grain yield and quality parameters, depending on the resistance of wheat varieties. 

During kernel development, protein is formed first, while starch synthesis and kernel fill occurs later. Thinner and high protein kernels have less extract (inverse relationship between protein and starch). We can conclude that higher losses for starch and low molecular weight subunits during *Fusarium* infection will make some varieties (Olimpija) less convenient for malting. Also, we observed that some varieties had higher losses among other varieties in spike length and starch, because the interruption of assimilate transport within the spike reduced the normal development of kernels, leading to shrunken and shriveled kernels and therefore to a reduced 1000 kernel weight and test weight. Since biosynthesis of storage proteins is being held earlier in grain-filling than in starch synthesis, it might be the case that starch content and protein composition are affected more severely than protein content, where gluten make a major proportion of the total wheat protein. In *Fusarium-*inoculated treatment some varieties increased protein content, suggesting that FHB does not affect grain biosynthesis processes but rather impacts the transport of assimilates caused by changes in the grain composition, which was previously concluded by Martin et al. [28]. The most FHB resistant varieties can be used for the crossing programs to improve the technological quality of the bread wheat, as was previously concluded for desired technological traits by other researchers [29]. Nevertheless, the best control option for *Fusarium* disease, when available, is using FHB resistant wheat varieties. As a result, we investigated the protein distribution of four winter wheat varieties with different FHB resistance in the grains and corresponding malt, under higher FHB pressure, as well as in natural infection in the field conditions. While the majority of work conducted on *Fusarium* in malting has been on barley, the use of wheat grains is also more and more important for the malting industry. In this investigation we showed that behavior of proteins after malting differed between susceptible and resistant varieties, where FHB susceptible varieties are inconvenient for malting, due to yield and quality losses (glutenins and its subfractions changes). Selection of FHB resistant varieties can be an effective method in selecting wheat varieties with good quality for malting.

## 3. Materials and Methods 

### 3.1. Field Trials 

The study was conducted on the four commercially grown Croatian winter wheat (*Triticum aestivum* L.) varieties Vulkan, Olimpija, Kraljica and Golubica grown during two consecutive seasons (2015/2016 and 2016/2017) in 7.56 m^2^ field experimental plots arranged in a complete randomized block design and located at the Experimental Station of Agricultural Institute Osijek (Osijek, Croatia, 45°32′ N, 18°44’ E). Vulkan is known as a high yielding variety with moderate quality, previously characterized as more FHB resistant [30], while Kraljica and Olimpija have a good quality with moderate resistance. Golubica is a high quality variety with moderate yield, previously characterized as being *Fusarium* susceptible [31]. To control seed-borne diseases, seeds were treated with Vitavax 200 FF (thiram + carboxin) at a rate of 200 mL 100 kg^−1^. In each year, one treatment in two replications was left to natural infection and one treatment in two replications was artificially inoculated using *Fusarium* spp. twice at two day intervals. No fungicides were applied during the two years of investigation to gain possible natural infection, which regularly appears every year depending on the weather conditions. Insecticides and herbicides were used as needed to keep trials free from weeds and aphids.

The major soil type in this region is a eutric cambisol (measured at the experimental station of Agricultural Institute Osijek, pH-KCl—6.25, humus—2.20%, K_2_O 37.70 mg 100 g^−1^, P_2_O_5_ 39.70 mg 100 g^−1^) with the average humus content 2.23%. To meet the winter wheat plant nutrient requirements, fertilization differed during the study (N:P:K 120-140:80-100:120-150 kg ha^−1^). The mean annual temperatures during the vegetation seasons 2015/2016 and 2016/2017 were 11.0 and 10.0 °C, respectively. The sum of annual precipitation during this period was 705.8 and 481.5 mm. respectively. Wheat was harvested during beginning of July 2016 and 2017, respectively, by combine harvester, upon which grain yield was measured and converted in dt ha^−1^. Whole plot was harvested and samples were taken for further analysis (from each replications separately). 

### 3.2. Inoculum Production, Inoculation and Disease Assessment

*Fusarium* inoculum was produced in the Phytopathological laboratory of Agricultural Institute Osijek. Spore cultures of *F. graminearum* (PIO 31, isolate from the field of eastern Croatia obtained from a single spore technique [32]) and *F. culmorum* (IFA 104, DON chemotype and highly aggressive isolate, obtained from Institute of Biotechnology, IFA-Tulln, Austria) were sub-cultured on synthetic nutrient-poor agar (SNA) medium. After ten days, the agar was cut into plugs and these were used for multiplication of spores. The required amount of inoculum (concentration 1 × 10^5^ mL^−1^) for *F. graminearum* was produced by the ‘bubble breeding method’ [33] by using mung bean medium and for *F. culmorum* production (concentration 1 × 10^5^ mL^−1^) was done with mixture of wheat and oat (3:1 in volume) [34]. Spray inoculations with both *Fusarium* spp. (1:1) were performed at the flowering stage (Zadok’s scale 65) [35] at late afternoon using a tractor back sprayer. Disease assessment began with the appearance of the first symptoms, 10 or 18 days after *Fusarium* inoculation treatment in 2016 and 2017, respectively, followed by four consecutive scores at intervals of four days which was used to calculate the area under disease progress curve (AUDPC). The percentage of bleached spikelets (disease intensity) per plot was estimated according to a linear scale (0%–100%). FHB intensity per plot was taken as a measure for general resistance (GR).

### 3.3. Agronomical and Physiological Traits

The spike length was measured in the field from the base of the spike to the tip, excluding awns. After threshing, kernels were weighed followed by determination of the grain yield and 1000 kernel weight. Wheat yields were standardized to 14% moisture. Indirect quality traits (test weight, protein and starch) were analyzed with Infratec 1241 Analyzer (ICC standard method No 105/2; No 155; No 116/1). Relative trait measure loss in both years in *Fusarium*-inoculated treatment was determined relative to naturally infected treatment. 

### 3.4. Malting

The malting was done in an Automated Joe White Malting Systems Micro-malting Unit (Perth, Australia) at the Agricultural Institute Osijek, Croatia by using 200 g of the grains. The process started with 37 h of steeping (16 °C, 5 h submerged; 17 °C, 12 h air rest with 100% airflow; 17 °C, 6 h submerged; 18 °C, 12 h air rest with 100% airflow; 17 °C, 2 h submerged) to increase the moisture of the grain to approximately 45%. The germination phase lasted 96 h (17 °C, 75% airflow, 1.5 turn every 2 h). At the end of program, 18 h of kilning occurred (60 °C, 6 h; 65 °C, 3 h; 68 °C, 2 h; 70 °C, 2 h; 80 °C, 2 h; 83 °C, 2 h; 85 °C, 1 h). Shoots and roots were removed and the malt was stored in plastic containers with caps at −20 °C until further analysis.

### 3.5. Protein Composition Analysis

Extracted wheat proteins from the grain and malt were analyzed according to the method of Wieser et al. [36] using high-performance liquid chromatography (HPLC) (Perkin Elmer Instruments, USA) coupled with Total-Chrom software and a photodiode array detector. Elution of AG, GLI and GLU was performed with linear gradient of acetonitrile (ACN/0.1%TFA) in the water (H_2_O/0.1%TFA) from 24–54% over 30 min at flow rate of 1 mL min^−1^ and column temperature of 50 °C. Proteins were separated on a C18 reverse phase column (5 μm, 4.6 × 150 mm; Sigma-Aldrich Chemie GmbH, Germany). Quantification of protein fractions was based on measuring its peak area at 210 nm. All the determinations were repeated twice. The peak areas under AG, GLI and GLU chromatograms were summed and used as a direct measure of total content of extractable wheat proteins. Consequently, the proportions (%) of protein fractions and single protein types were calculated. 

### 3.6. Data Analysis

For disease severity, the area under disease progress curve (AUDPC) of individual assessment data was calculated. The data obtained for FHB severity for non-inoculated plots were not included because they were not visually scored due to showing no visual symptoms. Data about protein composition in both treatments were statistically processed by Statistica version 13.1 (TIBCO Software, Palo Alto. CA, USA) with a level of significance set at α = 0.05. Differences between means were evaluated using the nonparametric Fisher-LSD test. Protein composition was expressed as means of two replications (±SE). Different letters indicated a significant difference between treatments (naturally-infected/inoculated) in varieties.

## 4. Conclusions

Our results showed that under higher pressure *Fusarium* Head Blight (FHB) infestation, the content of glutenins decreased in the susceptible variety Golubica. On the other side, the content of glutenins in resistant varieties was slightly reduced or unchanged. In the susceptible variety Golubica during *Fusarium*-inoculated treatment, compared to naturally-infected treatment, glutenins, and high and low molecular weight glutenin subunits were increased after malting in 2016. In 2017, when disease pressure was higher than in 2016, after malting, there was a tendency in all varieties to increase gliadins and its sub fractions, and to decrease glutenins and its sub fractions in *Fusarium*-inoculated treatment. 

High quality malt provides the color and flavor compounds which contribute to the final character of beer. Satisfactory wheat malt quality needs to have a good insoluble/soluble protein ratio, which was obtained by resistant varieties in *Fusarium*-inoculated samples, compared to during naturally-infected treatment. FHB induced significant yield and quality losses in susceptible *Fusarium* variety Golubica. Therefore, if wheat protein content is degraded in susceptible varieties, there may be insufficient enzymatic activity to modify the wheat grain and break down starch for brewing.

The majority of information on the impact of FHB disease on malting and brewing quality can be provided by artificially inoculated pre- or post-harvest experiments of wheat grain and malt.

## Figures and Tables

**Table 1:** Disease severity at 10, 14, 18, 22 and 26 days post inoculation (dpa) and the area under disease progress curve (AUDPC) calculation in *Fusarium*-inoculated treatment.

**2016**						
	10	14	18	22	26	AUDPC
Vulkan	0.00	0.0	1.0	4.0	7.5	18.3
Olimpija	0.00	0.0	1.5	7.5	17.5	38.3
Kraljica	0.00	0.0	2.5	6.5	20.0	44.0
Golubica	0.00	0.0	4.0	20.0	55.0	114.5
**2017**						
Vulkan	1.75	4.0	7.5	17.5	20.0	90.8
Olimpija	1.25	5.0	5.0	10.0	17.5	72.5
Kraljica	2.00	3.5	5.0	10.0	20.0	75.5
Golubica	4.25	15.0	52.5	75.0	87.5	430.0

**Table 2:** Relative losses in *Fusarium*-infected treatment, compared to naturally infected in two years data (2016 and 2017) for grain yield (GY) *, test weight (TW), 1000 kernel weight (TKW), spike length (SL), protein content (PRO) and starch (ST).

Relative Losses (%)						
**2016**						
Varieties	GY*	TW	TKW	SL	PRO	ST
Vulkan	10.3	0.8	5.0	−21.4	−0.4	2.2
Olimpija	38.5	3.0	8.7	0.0	−4.6	3.2
Kraljica	−13.0	−7.9	−20.4	16.7	−11.9	4.8
Golubica	37.0	15.4	−2.2	17.4	−1.5	4.1
**2017**						
Vulkan	15.6	2.7	6.6	5.9	−0.9	1.6
Olimpija	8.1	3.5	5.4	11.1	−1.0	7.1
Kraljica	25.6	4.0	16.6	0.0	0.6	2.2
Golubica	56.2	15.4	30.6	13.6	2.2	−2.6

## Supplementary Materials

The following are available online at https://www.mdpi.com/2076-0817/8/3/112/s1.
