# Supplementary material for: Changes in Protein Composition in the Grain and Malt after Fusarium Infection Dependently of Wheat Resistance
Source: Pathogens. 2019 Jul 26;8(3):112. doi: 10.3390/pathogens8030112 (PMC6789662; doi:10.3390/pathogens8030112)
Supplement: Supplementary file 1 [file pathogens-08-00112-s001.pdf]

Supplementary Material

Table S1. Mean square.

		Mean square							
Source of variation	Df	% AG[1]	% GLI	%ω-GLI	%α-GLI	%γ-GLI	% GLU	%HMW-GS	%LMW-GS
Variety (V)	3	94.99 ***	84.80 ***	38.89 ***	145.86 ***	36.00 ***	31.91 ***	21.88 ***	32.85 ***
Treatment (T)	1	1.24 *	507.50 ***	23.81 ***	302.86 ***	0.056 ns	555.82 ***	73.67 ***	235.58 ***
Year (Y)	1	33.84 ***	110.17 ***	9.63 ***	14.03 ***	8.17 ***	268.32 ***	6.87 ***	191.44 ***
Malt/grain (MG)	1	26.53 ***	1122.57 **	5.79 ***	532.48 ***	51.42 ***	1489.30 ***	141.64 ***	712.97 ***
Replication (R)	1	1.16 ns	0.10 ns	0.02 ns	0.78 ns	0.06 ns	0.08 ns	0.012 ns	0.53 ns
Error	31	0.20	0.24	0.12	0.31	0.33	0.23	0.06	0.11
